# Disclosure of the Molecular Mechanism of Wheat Leaf Spot Disease Caused by *Bipolaris sorokiniana* through Comparative Transcriptome and Metabolomics Analysis

**DOI:** 10.3390/ijms20236090

**Published:** 2019-12-03

**Authors:** Wei Ye, Taomei Liu, Weimin Zhang, Saini Li, Muzi Zhu, Haohua Li, Yali Kong, Liqiong Xu

**Affiliations:** State Key Laboratory of Applied Microbiology Southern China, Guangdong Provincial Key Laboratory of Microbial Culture Collection and Application, Guangdong Open Laboratory of Applied Microbiology, Guangdong Institute of Microbiology, Guangdong Acadamy of Sciences, Guangzhou 510070, China; yewei@gdim.cn (W.Y.); liutm@gddcm.com (T.L.); lisn@gdim.cn (S.L.); zhumz@gdim.cn (M.Z.); lihh@gdim.cn (H.L.); yaoyz@gdim.cn (Y.K.); xulq@gdim.cn (L.X.)

**Keywords:** *Bipolaris sorokiniana*, comparative transcriptome, pathogenicity, cell wall-degrading enzymes, toxin producing, defensive response

## Abstract

Wheat yield is greatly reduced because of the occurrence of leaf spot diseases. *Bipolaris sorokiniana* is the main pathogenic fungus in leaf spot disease. In this study, *B. sorokiniana* from wheat leaf (W-*B. sorokiniana*) showed much stronger pathogenicity toward wheat than endophytic *B. sorokiniana* from *Pogostemon cablin* (P-*B. sorokiniana*). The transcriptomes and metabolomics of the two *B. sorokiniana* strains and transcriptomes of *B. sorokiniana*-infected wheat leaves were comparatively analyzed. In addition, the expression levels of unigenes related to pathogenicity, toxicity, and cell wall degradation were predicted and validated by quantitative reverse transcription polymerase chain reaction (qRT-PCR) analysis. Results indicated that pathogenicity-related genes, especially the gene encoding loss-of-pathogenicity B (LopB) protein, cell wall-degrading enzymes (particularly glycosyl hydrolase-related genes), and killer and Ptr necrosis toxin-producing related unigenes in the W-*B. sorokiniana* played important roles in the pathogenicity of W-*B. sorokiniana* toward wheat. The down-regulation of cell wall protein, photosystem peptide, and rubisco protein suggested impairment of the phytosynthetic system and cell wall of *B. sorokiniana*-infected wheat. The up-regulation of hydrolase inhibitor, NAC (including NAM, ATAF1 and CUC2) transcriptional factor, and peroxidase in infected wheat tissues suggests their important roles in the defensive response of wheat to W-*B. sorokiniana*. This is the first report providing a comparison of the transcriptome and metabolome between the pathogenic and endophytic *B. sorokiniana* strains, thus providing a molecular clue for the pathogenic mechanism of W-*B. sorokiniana* toward wheat and wheat’s defensive response mechanism to W-*B. sorokiniana.* Our study could offer molecular clues for controlling the hazard of leaf spot and root rot diseases in wheat, thus improving wheat yield in the future.

## 1. Introduction

Wheat is an important global economic crop, and is the most widely cultivated plant worldwide. However, wheat yield is greatly reduced because of leaf spot and root rot diseases [1]. In addition, effective measures for controlling the diseases are not yet available. *Bipolaris sorokiniana* is known as a pathogenic fungus of wheat disease [2,3,4], causing a great loss of wheat yield in warm and humid wheat-growing areas, particularly China, the United States, and Africa [5]. The pathogenic mechanism of *B. sorokiniana* toward wheat remains to be clarified [6]. Therefore, it is of great significance to elucidate the pathogenic mechanism of *B. sorokiniana* toward wheat to control wheat disease and improve the wheat yield.

Plant endophytic fungi are microorganisms living in plant tissues that do not cause obvious symptoms of disease in plants [7]. Fungal taxa such as *Alternaria*, *Colletotrichum*, *Phoma*, *Phomopsis,* and *Xylaria* are the dominant plant endophytic fungi [7,8], and can produce various kinds of secondary metabolites [9,10,11], including cytotoxic compounds to reduce host plant performance and fitness slightly [12,13]. By contrast, some endophytic fungi, such as species of *Trichoderma*, can control the hazard of nematodes in crops, thus facilitating the growth of host plants [14,15]. The rice endophyte *Harpophora oryzae* and pathogenic *Magnaporthe oryzae* were reported to share a common pathogenic ancestor [16]. The transcriptome data of the two fungi indicated that most gene-encoding enzymes involved in the glycolysis and tricarboxylic acid cycle were suppressed in *H. oryzae*-challenged rice roots (Ho-roots); meanwhile, the expression levels of these enzymes were enhanced in *M. oryzae*-challenged rice roots (Mo-roots), resulting in the accumulation of glucose and fructose in Ho-roots, which was not detected in Mo-roots. This phenomenon partly illustrates the pathogenic mechanism of *M. oryzae* toward rice and the evolutionary relationship of pathogenic *M. oryzae* and nonpathogenic endophytic fungus *H. oryzae* [13,16,17,18]. The endophytic *H. oryzae* also evolved from the pathogenic endophytic fungus *M. oryzae*. *Phyllosticta capitalensis* is an endophyte and a weak plant pathogen with a worldwide distribution in 70 plant families [19], and *Phyllosticta capitalensis* was described by Hennings [20], who found it was associated with necrotic leaves of *Stanhopea* sp. (Orchidaceae) collected in Brazil, indicating some endophytic fungi showed pathogenicity under certain conditions. The much weaker pathogenicity of *B. sorokiniana* from *Pogostemon cablin* (P-*B. sorokiniana*) compared with *B. sorokiniana* from wheat (W-*B. sorokiniana*) in our investigation was probably due to the coexistence with its hosts, resulting in the different genetic characteristics which still need further investigation. The spore-formation-related genes, cell wall-degrading enzyme (CWDE)-related genes, and toxin-producing related genes were reported to play significant roles in the pathogenicity of pathogenic fungi. It has been reported that the *MPG1* gene encodes a kind of hydrophobic protein which is very important for the development of conidia in *M. grisea*, and the deletion of *MPG1* significantly reduced the pathogenicity of *M. grisea* toward rice [21,22]. The glycosyl hydrolase secreted by *Phytophthora sojae* attacks the cell wall during the invasion of *P. sojae* toward soybean [23]. Different kinds of toxins, including sesquiterpenoidal glucopyranoside toxins with strong cytotoxicity toward barley roots, were isolated from pathogenic *B. sorokiniana* [24], causing serious symptoms of root rot disease. The elucidation of the molecular pathogenic mechanism of *B. sorokinia* could provide a theoretical guide for the resistance of wheat infected by *B. sorokinia*. However, the detailed molecular pathogenic mechanism of *B. sorokiniana* towards wheat has not been reported systematically.

In our previous study, a strain of *B. sorokiniana* was isolated from the stem of *Pogostemon cablin,* and 10 cochlioquinones with cytotoxic activity were isolated from the fermentation liquid of this *B. sorokiniana* strain [25]. Cochlioquinones were reported to be associated with diseases in cereal crops [26]. However, as found in our previous work, the strain P-*B. sorokiniana* from *P. cablin* exhibited much weaker pathogenicity toward wheat but produced considerably more secondary metabolites compared with the strain of pathogenic *B. sorokiniana* from wheat leaf (W-*B. sorokiniana*). Thus, the transcriptomes of the two *B. sorokiniana* strains and the two *B. sorokiniana* strain-infected wheat leaves were sequenced using Illumina paired-end sequencing technology. The expression levels of unigenes related to the spore formation, the pathogenic protein, the degradation of cell wall, and the toxin-production in the two *B. sorokiniana* strains were predicted and validated by quantitative reverse transcription polymerase chain reaction (qRT-PCR). The metabolomic analysis of the P-*B. sorokiniana* and W-*B. sorokiniana* was also performed. This work is the first comparative transcriptome combined with metabonomic analysis of pathogenic and endophytic *B. sorokiniana* strains. Our investigation can provide clues for elucidating the molecular mechanism of the pathogenicity of *B. sorokiniana* toward wheat, as well as the molecular relationship between pathogenic and endophytic *B. sorokiniana* strains. Thus, our investigation would lay a theoretical foundation for controlling wheat diseases caused by *B. sorokiniana*.

## 2. Results

### 2.1. Pathogenicity of the Two B. sorokiniana Strains

**Figure 1:**
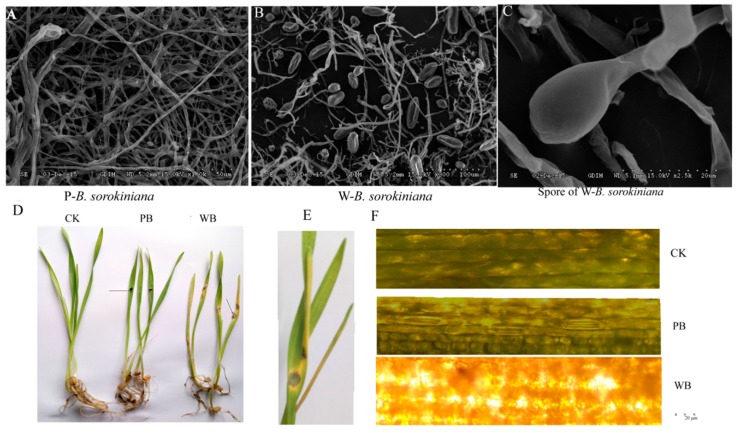
The pathogenicity of *Bipolaris sorokiniana* from *Pogostemon cablin* (P-*B. sorokiniana*) and *B. sorokiniana* from wheat leaf (W-*B. sorokiniana*): (**A**) The scanning electron microscope (SEM) image of mycelia of P-*B. sorokiniana*; (**B**) The SEM image of mycelia and spores of W-*B. sorokiniana*; (**C**) The SEM image of a spore of W-*B. sorokiniana*; (**D**) The wheat challenged by sterilized water, P-*B. sorokiniana*, and W-*B. sorokiniana*, respectively. The arrows indicate the place where the wheat leave is challenged with *B. sorokiniana* strain; (**E**) The symptoms of wheat leaves challenged by W-*B. sorokiniana;* (**F**) The optical microscope images of wheat leaves challenged by sterilized water, P-*B. sorokiniana*, and W-*B. sorokiniana*, respectively. CK, PB, and WB refer to uninfected wheat, P-*B. sorokiniana*-infected wheat, and W-*B. sorokinana*-infected wheat, respectively.

The strains of P-*B. sorokiniana* and W-*B. sorokinana* were cultured on PDA medium at 28 °C for seven days and observed by scanning electron microscopy (SEM). As shown in Figure 1, many spores were observed in pathogenic W-*B. sorokiniana*, while no spores were observed in endophytic P-*B. sorokiniana* (Figure 1A–C). Meanwhile, only very slight symptoms (very small disease spots, which were too small to be measured, more yellow in color than the CK group (uninfected wheat), with a slightly loose cell wall) were observed in the group inoculated with P-*B. sorokiniana* (PB), and the average length of the wheat leaves of the PB group was 12.05 ± 1.28 cm, which was only a little shorter than that in the CK group (12.8 ± 1.08 cm). Much shorter (10.62 ± 1.35 cm) and more yellow leaves (Figure 1D,E), black disease spots (with an average diameter of 0.68 ± 0.05 cm), and a degraded cell wall (Figure 1F) were observed in the group challenged by W-*B. sorokiniana* (WB). Thus, the disease symptoms of wheat infected by P-*B. sorokinana* were identified as grade 1, and the disease symptoms of wheat infected by W-*B. sorokinana* were identified as grade 6 according to the method of Fetch and Steffenson [27]. The infected wheat leaves were separated and cultured on PDA medium based on the isolation and cultivation of pathogen, and the procedure of Koch’s postulation. Thus, the obvious disease spots on wheat were determined to be caused by W-*B. sorokiniana*, which showed much stronger pathogenicity toward wheat than P-*B. sorokiniana*.

### 2.2. The Metabolomics Analysis of the Two B. sorokiniana Strains

**Figure 2:**
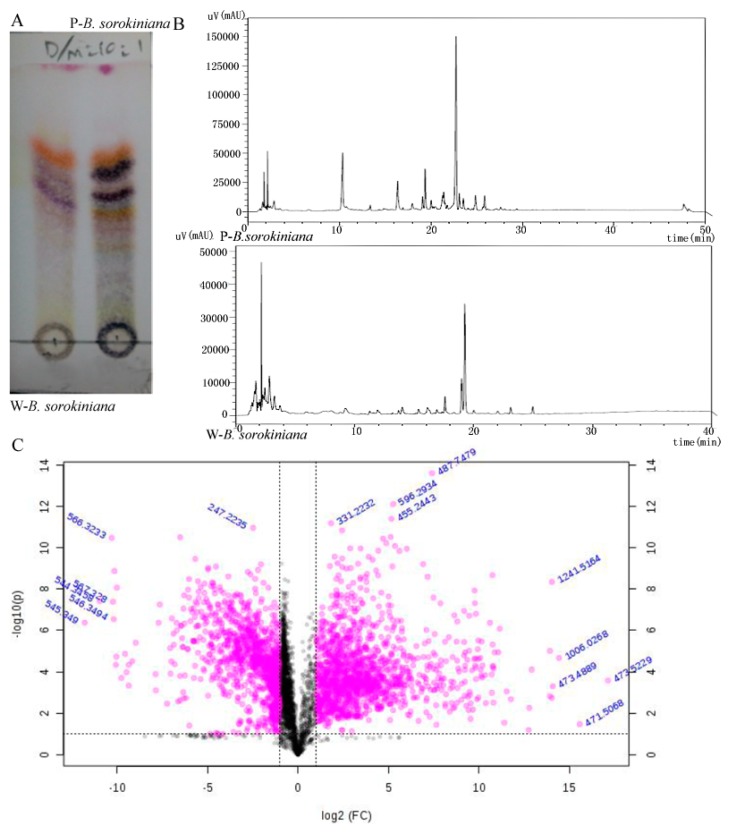
The detection of secondary metabolites of P-*B. sorokiniana* and W-*B. sorokiniana* strains: (**A**) TLC detection, the color lines indicate different kinds of secondary metabolites; (**B**) HPLC detection; (**C**) The volcano plot of differential metabolites between P-*B. sorokiniana* and W-*B. sorokiniana* strains. The red dots represent the differential fold of the two *B. sorokiniana* strains were more than 2 fold, and *P* < 0.05, the black dots represent the differential fold of the two *B. sorokiniana* strains were less than 2 fold, and *P* > 0.05. The dotted lines indicate the boundary between the red dots and the black dots.

The metabolites of P-*B. sorokiniana and* W-*B. sorokiniana* strains from fermentation liquids were extracted using ethyl acetate and detected by thin-layer chromatography (TLC) (Figure 2A) and high-performance liquid chromatography (HPLC) at 205 nm (Figure 2B). More bands were observed in the extract of P-*B. sorokiniana* by TLC detection, and more peaks were observed in the extract of P-*B. sorokiniana* than in that of W-*B. sorokiniana*. The results indicated the presence of more kinds of secondary metabolites in P-*B. sorokiniana* than in W-*B. sorokiniana.*

The extracts of fermentation liquids of the two *B. sorokiniana* strains were loaded to LC-MS, and the metabolomic analysis was performed, the differential secondary metabolites (>2-fold, *p* < 0.05) are shown in the volcano plot (Figure 2C).

The pathway enrichment of the differential metabolites is shown in Table S1. The results indicated the most differential metabolites (three metabolites) hit the pathway of vitamin A and aminoacyl-tRNA metabolism, and the hit numbers of differential metabolites in the pathways including vitamin B6 metabolism, thiamine metabolism, beta-alanine metabolism, pantothenate and CoA biosynthesis, phenylalanine, tyrosine, and tryptophan biosynthesis, arginine and proline metabolism, pyrimidine metabolism, and purine metabolism took second place (two metabolites). The significant differential secondary metabolites (*P* < 0.05) between the two *B. sorokiniana strains* hit vitamin B6 metabolism; thiamine metabolism and beta-alanine metabolism took second and third place, respectively.

### 2.3. Annotation of the Assembled Unigenes in Transcriptomes of B. sorokiniana-Infected Wheat

The endophytic strain P-*B. sorokiniana* was cultivated on potato dextrose agar (PDA) medium at 28 °C for three days (samples A3-1 and A3-2) and seven days (samples A7-1 and A7-2), respectively, and the pathogenic W-*B. sorokiniana* isolated from infected leaf tissues was also cultivated on PDA medium at 28 °C for seven days (samples B7-1 and B7-2). Then, these samples were collected and transcriptome sequenced. All the raw data were deposited in National Center for Biotechnology Information (NCBI) with accession number SRR5007101. A total length of 196,685,628 clean reads with a Q20 value of higher than 98.5% and high-quality clean read ratio higher than 98.0% were obtained with an average length of 1047 bp. This result indicated the high quality of the transcriptome sequencing. Accordingly, 20,434, 20,289, and 24,073 unigenes were assembled in the A3, A7, and B7 groups, respectively. Up to 26,460 unigenes were annotated in databases including Nr, SwissProt, euKaryotic Orthologous Groups (KOG), and Kyoto Encyclopedia of Genes and Genomes (KEGG) (Figure S1). A total of 7086 unigenes were not annotated. Approximately 2891 unigenes were annotated as pathogen-related genes in the pathogen–host interaction database. In total, 1065 unigenes could match the PHI database from fungi colonizing/pathogenic on wheat. As shown in Figure S2A, all the groups (especially A7 and B7) showed very high repeatability. The transcriptome of *B. sorokinana*-infected groups including unchallenged wheat (CK), P-*B. sorokiniana*-treated wheat (PB), and W-*B.sorokinana*-treated wheat (WB) groups were sequenced and annotated based on known databases and transcriptome of P-*B. sorokinana* and W-*B. sorokinana*. The PCA analysis of the CK, PB, and WB groups also showed high repeatability (Figure S2B), demonstrating the reliability of the transcriptome results.

**Figure 3:**
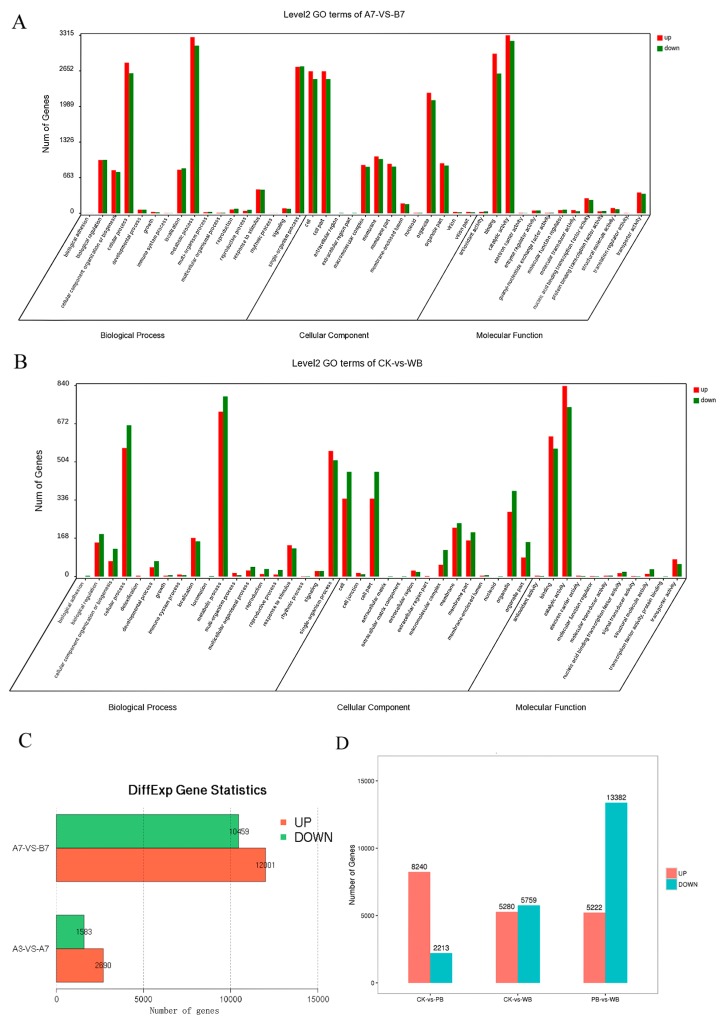
Gene Ontology (GO) enrichment of the *B. sorokiniana* transcriptome. (**A**) Differential GO enrichment between the A7 and B7 transcriptomes. (**B**) Differential GO enrichment between transcriptomes of the CK and WB groups. (**C**) Differential unigenes between transcriptomes of the A3, A7, and B7 groups. (**D**) Differential unigenes between transcriptomes of the CK, PB, and WB groups.

The length distribution of *B. sorokiniana* transcriptome is shown in Figure S3. All the unigenes were classified into 25 KOG functions (Figure S4). The Gene Ontology (GO) enrichment results showed that group of unigenes annotated with functions in catalytic activity, the metabolic process, and the cellular process showed the greatest differential expression between the A7 vs. B7 group and the CK vs. WB group, respectively. The comparative transcriptome results showed that 2690 unigenes were up-regulated and 1583 unigenes were down-regulated in the A3 vs. A7 group. Meanwhile, up to 12,001 unigenes were up-regulated and 10,459 unigenes were down-regulated in the A7 vs. B7 group (Figure 3C). In total, 8240 unigenes were up-regulated and 2213 unigenes were down-regulated in the CK vs. PB group, and up to 5280 unigenes were up-regulated and 5759 unigenes were down-regulated in the CK vs. WB group (Figure 3D), which was probably due to the presence of more versatile secondary metabolites in P-*B. sorokiniana* as compared to W-*B. sorokiniana.*

**Figure 4:**
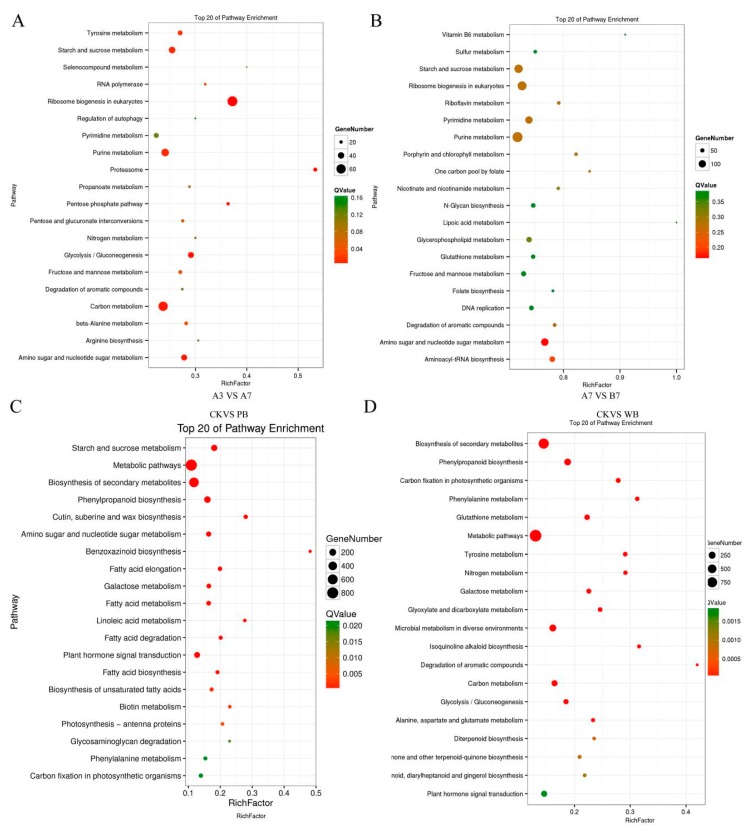
Kyoto Encyclopedia of Genes and Genomes (KEGG) pathway enrichment of the differential unigenes between transcriptomes of *B. sorokiniana* strains. (**A**) The A3 group and A7 group. (**B**) The A7 group and B7 group. (**C**) The CK group and PB group. (**D**) The CK group and WB group.

The pathway enrichment results of the A3 vs. A7 group indicated that the ribosome biogenesis pathway showed the most significant difference. The proteasome pathway took second place (Figure 4A). While the amino sugar and nucleotide sugar metabolism pathway showed the most significant difference between the A7 vs. B7 group, the aminoacyl-tRNA biosynthesis pathway took second place (Figure 4B).

In total, 86,784, 91,294, and 83,896 genes were annotated in the transcriptomes of the CK, PB, and WB groups, respectively. Overall, 5280 and 5759 genes were up-regulated and down-regulated in the WB group compared with CK group, respectively. It was found that 8240 and 2213 genes were up-regulated and down-regulated in the PB group compared to the CK group, respectively. The significant differential genes of the CK vs. PB, and CK vs. WB groups were enriched in the KEGG pathway. The starch and sucrose metabolism took first place between the CK group and PB group (Figure 4C). The biosynthesis of secondary metabolites took first place between the CK group and WB group (Figure 4D). Phenylpropanoid biosynthesis took second place. Phenylpropanoid biosynthesis pathway is reported to be responsible for the resistance to fungal infection and pigment formation.

### 2.4. The Differential Expression of Pathogenicity-Related Unigenes

**Figure 5:**
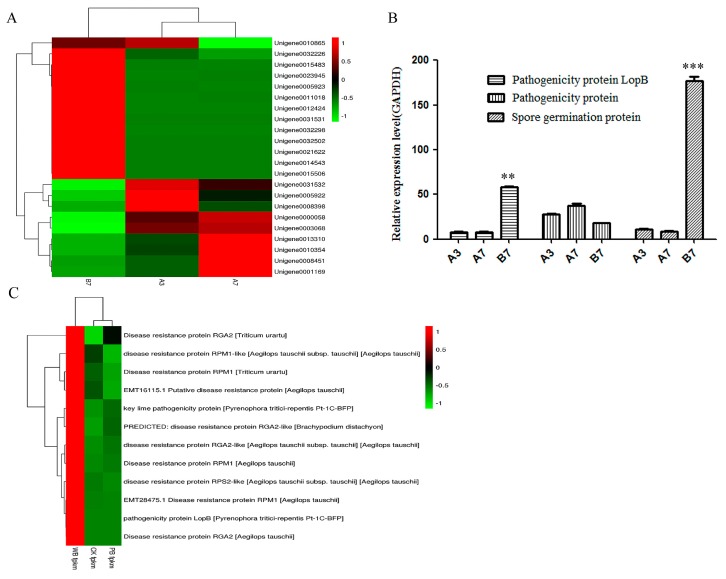
The expression levels of unigenes related to the pathogenicity protein and spore germination protein. (**A**) A heatmap of unigenes related to the pathogenicity protein and spore germination protein of the A3, A7, and B7 groups. (**B**) qRT-PCR analysis. Pathogenicity protein LopB and spore germination protein showed significantly higher expression levels in W-*B. sorokiniana* than in P-*B. sorokiniana*. (**C**) A heatmap of unigenes related to the pathogenicity protein and spore germination protein of the CK, PB, and WB groups. ** p < 0.05, *** *p* < 0.001.

A contradictory phenomenon was observed in W-*B. sorokiniana* considering that no spores and very low pathogenicity were observed in P-*B. sorokiniana*. Thus, the expression levels of unigenes related to pathogenicity were predicted. The GO terms of all unigenes from the two *B. sorokiniana* strains are annotated in Table S2. As shown in Figure 5A, most unigenes related to pathogenicity showed a higher expression level in the B7 group than in the A7 and A3 groups. Moreover, more unigenes related to pathogenicity and spore formation showed higher expression levels in the A7 group than in the A3 group. In addition, three unigenes involved in the pathogenicity and spore germination were validated by the results of qRT-PCR. The expression level of unigene 0005923 encoding the pathogenicity protein LopB in W-*B. sorokiniana* was approximately 8-fold higher as compared to P-*B. sorokiniana* (Figure 5B). No significant difference was observed in the expression level of another pathogenicity protein. The expression level of spore formation protein (unigene0021622) was approximately 18-fold higher in the B7 group as compared to the A7 and A3 groups (Figure 5B). This effect was in accordance with the fact that many spores were observed in W-*B. sorokiniana* and no spores were observed in P-*B. sorokiniana*. The results indicated that the pathogenicity-related protein and spore formation-related protein have a close relationship with the pathogenicity of *B. sorokiniana*.

Three unigenes encoding pathogenicity-related proteins were found in the transcriptomes of CK, PB and WB groups. According to the prediction result based on the fragments per kilobase million (FPKM) value, the pathogenicity protein loss-of-pathogenicity B (LopB) and key lime pathogenicity protein showed significant higher expression levels in the WB group than in the CK and PB groups, in accordance with the results of transcriptomes of the A3, A7, and B7 groups. Moreover, all the 16 unigenes encoding disease-resistance protein showed significantly higher expression levels in the WB group as compared to the CK and PB groups (Figure 5C).

### 2.5. Unigenes Encoding Cell Wall-Degrading Enzymes

**Figure 6:**
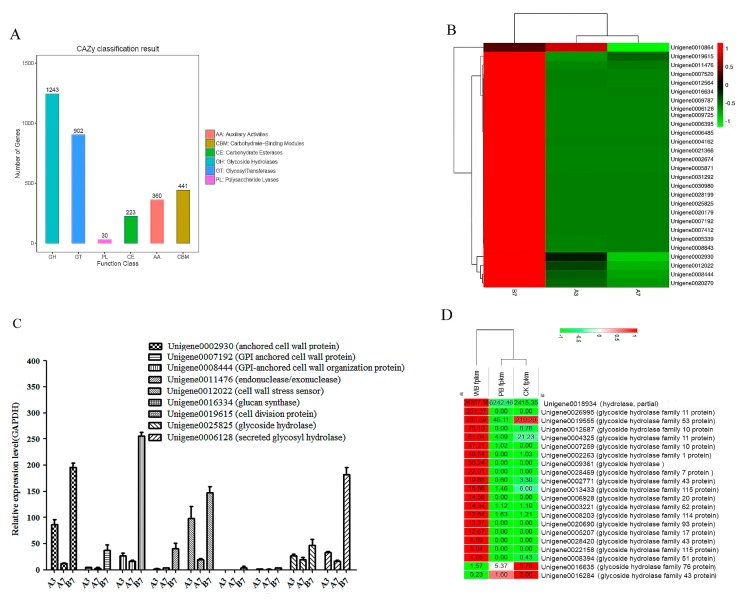
The expression levels of unigenes related to cell wall-anchored proteins and cell wall-degrading enzymes. (**A**) classification of carbohydrate-active enZYmes (CAZYs) in *B. sorokiniana*. (**B**) The heatmap of unigenes related to the cell wall degradation. (**C**) qRT-PCR analysis. (**D**) The heatmap of unigenes related to cell wall-anchored proteins and cell wall-degrading enzymes of the CK, PB, and WB groups.

The unigenes of the two *B. sorokiniana* strains are annotated in the carbohydrate-active enZYmes (CAZYs) database (Figure 6A). Most CAZYs were annotated as glycoside hydrolases, and glycoside transferases took second place. The two kinds of CAZYs played important roles in the pathogenicity of different phytopathogenic fungi. Because the CWDEs play very important roles during infection, the expression level of unigene encoding CWDE was also investigated. According to the heatmap of CWDE, most anchored cell wall proteins and CWDEs showed higher expression levels in the B7 group compared with the A3 and A7 groups. Only unigene 0010864 showed a higher expression level in the A3 group than in the B7 group. Unigene 0006128 encoding the important CWDE glycosyl hydrolase showed no expression in the A3 and A7 groups, whereas a relatively high expression level of glycosyl hydrolase was detected in the B7 group (Figure 6B). Moreover, nine significant differentially-expressed unigenes related to CWDEs were selected for qRT-PCR analysis. The qRT-PCR result showed nearly the same trend as the prediction. However, the differences of the expression levels of CWDEs between the different groups were not as significant as in the prediction (Figure 6C). In addition, most CWDEs showed slightly higher expression levels in the A3 as compared to the A7 group, suggesting the degradation of some CWDEs occurred during the growth of P-*B. sorokiniana*. The annotation of unigenes related to the cell-wall degrading is shown in Table S3. The cell-wall degradation of wheat leaves after being infected by W-*B. sorokiniana* indicated the important roles of anchored cell wall proteins and CWDEs during the infection of W-*B. sorokiniana* toward wheat leaves. The qRT-PCR analysis results showed that the expression level of unigene0006128 encoding glycosyl hydrolase showed nearly the same expression level as that in W-*B. sorokiniana* cultured on PDA medium, whereas no expression of glycosyl hydrolase was detected in the CK group and in the P-*B. sorokiniana*-challenged group (Figure 6C), demonstrating the important role of unigene 0006128 during the infection of W-*B. sorokiniana* toward wheat.

Twenty-one unigenes encoding glycosyl hydrolases were found in the CK, PB, and WB groups, as shown in Figure 6D. Among them 18 unigenes exhibited significant higher expression levels in the WB group as compared to the PB and CK group according to the FPKM values. The results indicated that unigene0016934 encoding hydrolase, unigene0026995 encoding glycoside hydrolase family 11 protein, and unigene0012687 encoding glycoside hydrolase family 10 protein played important roles in the pathogenicty of W-*B. sorokinina* toward wheat.

### 2.6. Unigenes Related to Toxin Production

**Figure 7:**
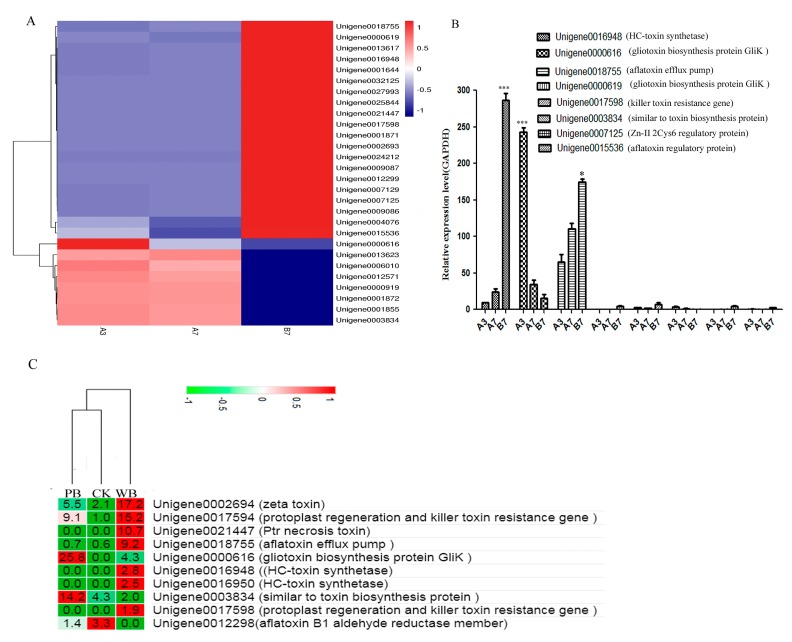
The expression levels of unigenes related to toxin-production in *B. sorokiniana* strains. (**A**) A heatmap of unigenes related to toxin-producing in the A3, A7, and B7 groups. (**B**) qRT-PCR analysis. (**C**) A heatmap of unigenes related to toxin-production in the CK, PB, and WB groups.

Toxins play a very important role for the pathogenicity of fungi. Thus, the expression levels of unigenes related to toxin production were investigated. In addition, unigenes related to the biosynthesis and metabolic process of gliotoxin, killer toxin, zeta toxin, *Helminthosporium carbonum* (HC) toxin, aflatoxin B1, and *Alternaria alternata* toxin were found in the transcriptome of *B. sorokiniana*. Most toxin-producing unigenes showed much higher expression levels in the B7 group than in the A3 and A7 groups. Unigenes 0001872, 0006010, and 0012571 related to the metabolic process of toxins showed higher expression levels in the A3 and A7 groups than in the B7 group, facilitating the metabolism of toxins in P-*B. sorokiniana*. Unigene 0001872 was annotated with the subject ID of Q2I0M6_CERNC in the pathogen-host interactions (PHI) database, which was an important gene for leaf disease spots caused by *Cercospora nicotianae*. This phenomenon resulted in the lower pathogenicity of P-*B. sorokiniana* compared with that of W-*B. sorokiniana*. In addition, the killer toxin resistance gene (unigene 0017598) showed a high expression level in W-*B. sorokiniana*, playing an important role in the tolerance of W-*B. sorokiniana* to various toxins (Figure 7A). Unigene 0017598 was annotated as an important gene for rice blast caused by *M. oryzae* in the PHI database. Eight differentially-expressed unigenes related to toxin-production were selected for qRT-PCR validation. The qRT-PCR results showed that most unigenes showed high expression levels in W-*B. sorokiniana*, but the differences between the expression levels in different groups were not significant, as predicted (Figure 7B).

The annotation of unigenes related to toxin-production is shown in Table S4. Most enzymes involved in the biosynthesis of aflatoxin were also down-regulated in the P-*B. sorokiniana* strain compared with the W-*B. sorokiniana* (Figure S5), leading to the production of more toxins and a higher pathogenicity in W-*B. sorokiniana*. Ten toxin-producing related uingenes were found in the CK, PB, and WB groups, and seven unigenes including those related to the biosynthesis of zeta toxin, HC toxin, killer toxin, and Ptr necrosis toxin showed significantly higher expression levels in the WB group as compared to the CK and PB groups, which was in accordance with the comparative transcriptomic and metabolomics analysis results of the A3, A7, and B7 groups. The results suggested the important roles of zeta toxin, H killer toxin, aflatoxin, Ptr necrosis toxin, and HC toxin in the pathogenicity of W-*B. sorokiniana* toward wheat (Figure 7C).

Moreover, a metabolomic analysis of the two *B. sorokiniana* strains cultivated at 28 °C for 7 days was performed. More than two-fold differences between the two groups are listed in Table S5. The metabolomics analysis results showed that the abundance of that anatoxin secreted by P-*B. sorokiniana* was only 0.479-fold that in W-*B. sorokiniana*. Meanwhile, the abundances of the main intermediate products in aflatoxin biosynthesis, versiconal and 6-demethylsterigmatocystin in P-*B. sorokiniana* fermentation liquid, were only 0.155-fold and 0.063-fold as compared to W-*B. sorokiniana*, respectively, in accordance with the significantly higher expression levels of toxin-producing unigenes in W-*B. sorokiniana* compared with P-*B. sorokiniana*.

### 2.7. The Validation of Differential Unigenes in CK, PB and WB Groups

**Figure 8:**
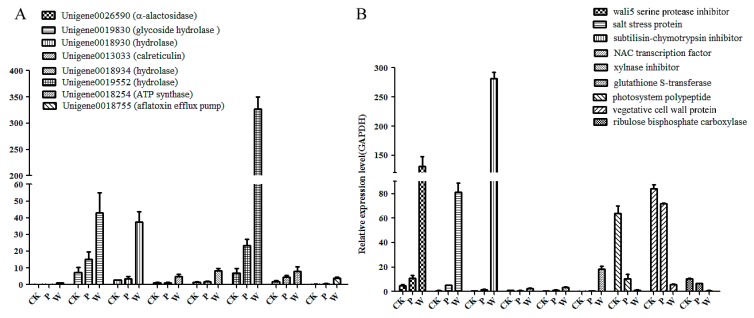
The qRT-PCR analysis of significant differential unigenes in the CK, PB, and WB groups. (**A**) the significant differential unigenes based on transcriptome of *B. sorokiniana*. (**B**) the significant differential unigenes related to wheat responding to *B. sorokiniana* infection.

Different primers were designed to perform qRT-PCR analysis in order to validate the significant differential unigenes in CK, PB, and WB groups. Seventeen significant differential genes in the WB group compared with the CK and PB groups were confirmed by qRT-PCR (Figure 8). The most differential unigenes in the WB, PB, and CK groups annotated based on the transcriptome database of the A3, A7, and B7 groups were validated by qRT-PCR. The most up-regulated genes in the WB group were unigenes encoding a-alactosidase, hydrolase, glycoside hydrolase, calreticulin, ATP synthase, and the aflatoxin efflux pump (Figure 8A). The results confirmed that these unigenes played key roles in the pathogenicity of W-*B. sorokiniana* toward wheat.

The most significantly up-regulated genes in the WB group were those genes encoding the serine protease inhibitor, salt stress protein, subtilisin-chymotrypsin inhibitor, NAC transcription factor, xylanase inhibitor, glutathione S-transferase, the unigene encoding subtilisin-chymotrypsin inhibitor, salt stress protein and *wali5* gene encoding serine protease inhibitor in the WB group, displayed more than 25-fold higher expression levels as compared to the CK group. The down-regulated genes in the WB group compared with PB and CK group were unigenes encoding photosystem polypeptide, vegetative cell wall protein, and ribulose biphosphate carboxylase (Figure 8B), which is responsible for the fixation of CO_2_ in the photosynthetic system, indicating that the photosynthetic system and ribosome of wheat might be impaired by the infection of W-*B. sorokiniana*. The up-regulation of pathogenesis-related protein PRB1-2-like in W-*B. sorokiniana*-infected wheat proved its function in the pathogenicity of W-*B. sorokiniana* toward wheat. The KEGG map of photosynthesis, oxidative phosphorylation, and peroxisome between the CK and WB group indicated that almost all unigenes related to photosynthesis were down-regulated in the WB group (Figure S7A), and the unigenes related to oxidative damage including cyclooxygenase-2 (COX2) were up-regulated in WB group (Figure S7B), indicating reactive oxygen species (ROS) were produced in W-*B. sorokiniana*-treated group. Meanwhile, most unigenes encoding peroxidase were up-regulated (Figure S7C) to remove excess ROS to protect the wheat cells against the damage of oxidation.

## 3. Discussion

In this study, we found that the endophytic *B. sorokiniana* strain from *P. cablin* and pathogenic strains isolated from infected wheat leaf, respectively, showed significantly different pathogenicities toward wheat. A comparative metabolomic analysis of the two *B. sorokiniana* strains was undertaken, and the comparative transcriptomes between the two *B. sorokiniana* strains as well as *B. sorokiniana* strain-infected wheat leaves were analyzed to investigate the molecular mechanism of the pathogenicity of *B. sorokiniana* and the relationship between the two different *B. sorokiniana* strains.

In our previous study, more toxins were detected in the fermentation liquid of W-*B. sorokiniana* compared with P-*B. sorokiniana* based on the metabonomic analysis, and most unigenes related to the biosynthesis of toxins showed much higher expression levels in W-*B. sorokiniana* than those in P-*B. sorokiniana*, which contributed to the stronger pathogenicity of W-*B. sorokiniana* compared with P-*B. sorokiniana*. In addition, most unigenes related to the spore formation, cell wall anchor, cell wall degradation, and toxin production showed higher expression levels in W-*B. sorokiniana* than those in P-*B. sorokiniana*. The findings indicated that these unigenes played very important roles in the pathogenicity of W-*B. sorokiniana*.

Up to 33,541 unigenes of the A7 and B7 groups were compared, and 22,460 unigenes with significantly different expression levels were found. A total of 11,081 unigenes did not show significant differences between the groups A7 and B7. This might be due to the different growth environments of P-*B. sorokiniana* and W-*B. sorokiniana*, which affected the expression of pathogenesis related-genes in *B. sorokiniana* strains. P-*B. sorokiniana* did not show strong pathogenicity toward *P. cablin*; we assumed that this strain may exist in symbiosis with *P. cablin*, a medicinal plant, which may endow the endophytic fungus P-*B. sorokiniana* with the feature of producing bioactive metabolites [11,25], thus resulting in more unigenes involved in the biosynthesis of other bioactive metabolites in P-*B. sorokiniana*. Moreover, fewer unigenes were involved in the pathogenicity in P-*B. sorokiniana* compared with W-*B. sorokiniana*, leading to the weak pathogenicity of P-*B. sorokiniana*.

Previous studies showed that the spores, cell-degrading enzymes, and toxins played significant roles in the pathogenicity of fungi [28]. The spores of *Fusicladium* sp. and *Collectotrichum lindemuthianum* can form appressorium adhering to plants, facilitating the invasion of fungi into host plants [29]. *Fusarium graminearum* was reported to produce cell-degrading enzymes, including cellulase, xylanase, and penctinase, facilitating the decomposition and loosening of cell walls and promoting the infection and expansion of *F. graminearum* in hosts [30,31,32]. The very loose cell wall structure of wheat leaves after being infected by W-*B. sorokiniana* suggested the important role of cell wall-anchored proteins and cell wall-degrading enzymes during the process of infection of wheat leaves by W-*B. sorokiniana*. In addition, *F. graminearum* can produce various kinds of trichothecene toxins, with strong cytotoxicity [33]. The mutated *F. graminearum* strain that cannot produce trichothecenes showed much weaker pathogenicity than native the *F. graminearum* strain with the ability to produce trichothecenes [34,35]. The spores, cell-degrading enzymes, and toxins played very important roles in the pathogenicity of *F. graminearum* strain. Our comparative transcriptome results of the two *B. sorokiniana* strains and the wheat leaves infected with two *B. sorokiniana* strains also demonstrated the important roles of unigenes related to spore formation, pathogenicity protein, cell-degrading enzymes, and toxin production in W-*B. sorokiniana* toward wheat. Most CWDEs involved in the biosynthesis of *N*-glycans were down-regulated in the P-*B. sorokiniana* strain compared with W-*B. sorokiniana*. The *N*-glycan biosynthesis pathway also displayed significant difference between the A7 and B7 groups (Figure S6A). It was reported that glycosyl hydrolases, namely BsGH7-3, with high stability played important role in the strong pathogenicity of *B. sorokiniana* toward wheat [36]. Most enzymes involved in the aflatoxin biosynthesis in P-*B. sorokiniana* were down-regulated compared with W-*B. sorokiniana* according to the KEGG annotation of the two *B. sorokiniana* strains (Figure S5), which was in accordance with the qRT-PCR results (Figure 7).The *fmk1* gene encoding a mitogen-activated protein kinase (MAPK) of *Fusarium oxysporum* was reported to be essential for root penetration and pathogenesis toward tomatoes [37]. The mutants of *F. oxysporum* carrying an inactivated copy of *fmk1* mutants lost pathogenicity in tomato plants. MAPK signaling pathway was also reported to be involved in phenotype switching and the formation of mycelia and spores, which played an important role in the invasive infection of *Candida albicans* [37]. The gene *stel2* was reported to be closely related with the hyphae formation and mitogen-activated protein kinases of the high-osmolarity glycerol (HOG1), which was strongly associated with pathogenicity of *C. albicans* [38]. The gene *stel2* was down-regulated in P-*B. sorokiniana* and HoG1 was up-regulated in W-*B. sorokiniana* according to the heap map analysis of the two *B. sorokiniana* transcriptomes (Figure S7A) and the KEGG map of MAPK pathway of the two *B. sorokiniana* strains (Figure S7B), which was in accordance with stronger pathogenicity of W-*B. sorokiniana* toward wheat than P-*B. sorokiniana*.

The comparative transcriptome analysis of the wheat leaves infected with the two *B. sorokiniana* strains indicated that the hydrolases, toxin-producing related unigenes, and defensive reponse-related unigenes including *gst* played important roles in the pathogenicity of W-*B. sorokiniana* and in the defensive response of wheat to *B. sorokiniana*, respectively. As hydrolases, glycosyl hydrolases in particular showed much higher expression levels in W-*B. sorokinana* than in P-*B. sorokinana*. Hydrolase inhibitor-related genes including xylnase inhibitor and subtilisin-chymotrypsin inhibitor displayed much higher expression levels in W-*B. sorokinana*-treated tissues to reduce the enzymatic activities of hydrolases enzymes, thus defending pathogenic factors of W-*B. sorokinana*. *Wali5* gene encodes the serine protease inhibitor, which is important for defense from the attack of pathogenic fungi by inhibiting the enzymatic activity of hydrolases. The *wali1* and *wali5* genes were highly expressed in shoot and root tissues of wheat by the induction of various abiotic stresses as well as in response to aluminum, plant hormones, and oxidative molecules [38]. NAC transcription factors could promote abiotic stress tolerance with respect to salt and iron-deficiency, salt stress, and biotic stress tolerance including pathogen infection in *Arabidopsis*, wheat, rice, and soybean [13,39,40]. The glutathione S-transferase (GST) has been demonstrated as an effective antidote for the excess reactive oxygen species in plants [41]. 

**Figure 9:**
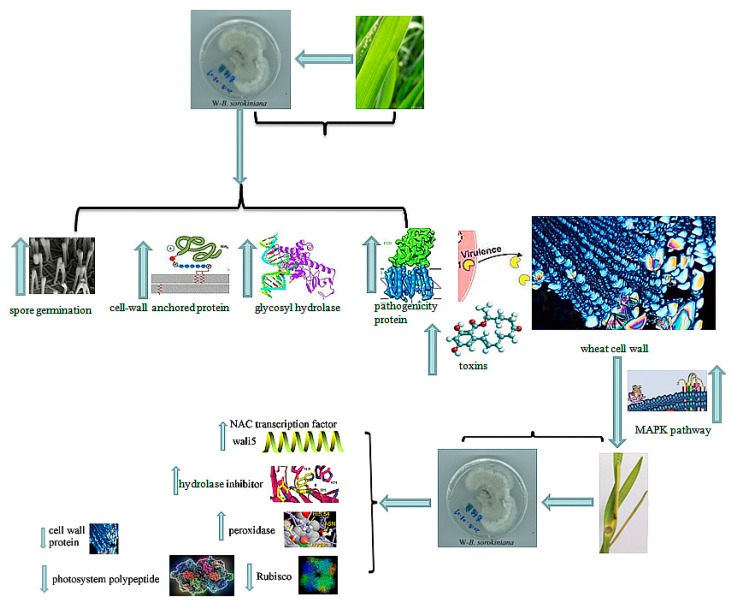
The proposed pathogenic mechanism of W-*B. sorokiniana* toward wheat and the defensive response of wheat. The upward arrow indicated the up-regulated genes in *B-sorokiniana* treated wheat compared with CK group. The downward arrow indicated the down-regulated genes in *B-sorokiniana* treated wheat compared with CK group.

The molecular pathogenic mechanism of W-*B. sorokiniana* toward wheat and wheat’s defensive responsive mechanism were proposed in this study (Figure 9). The spores of W-*B. sorokiniana* facilitate the adhesion and invasion of W-*B. sorokiniana* toward wheat leaves. The expression of unigenes encoding pathogenicity proteins, cell wall-anchored proteins, and cell wall-degrading enzymes including glycosyl hydrolases being up-regulated, and the expression of photosystem peptide and ribulose biphosphate carboxylase were down-regulated, thus impairing the photosynthetic system and cell wall structure of wheat leaves. Then, the MAPK pathway was activated, further facilitating the spore germination and the up-regulation of toxin-producing unigenes to promote the production of toxins and ROS, thereby causing DNA and protein damage in wheat leaves and finally forming the disease symptoms of wheat leaves. To reduce the hazard of ROS toward wheat leaves, the expression of pereoxidases including GST was up-regulated, and the *wali5* gene and NAC transcription factor were up-regulated to enhance the biotic stress tolerance of wheat leaves infected by W-*B. sorokiniana.* The hydrolase inhibitors were also up-regulated to protect the cell wall of wheat leaves from the attack by hydrolases, especially glycosyl hydrolases produced by W-*B. sorokiniana.*

## 4. Materials and Methods

### 4.1. Plant Materials, Strains

Medicinal plant *P. cablin* was collected from Yangchun City, Guangdong Province, China. The endophytic *B. sorokiniana* (Accession No. KF494823.1) was isolated from the medicinal plant *P. cablin* (numbered as A606) [25]. The pathogenic *B. sorokiniana* isolated from wheat leaf (ACCC36514) was purchased from the Agricultural Culture Collection of China (Accession No. MF594383), collected from wheat leaf in Lanzhou City, Gansu Province. The susceptible wheat cultivar Jimai 20 (Shandong province, China) was used for all plant pathogenicity assays.

### 4.2. Pathogenicity Testing of the Two B. sorokiniana Strains

The wheat seed was cultivated in phytochamber at 25 °C with 60% humidity for seven days, with a photoperiod of 16 h. Then, the wheat leaves were soaked in 75% ethanol for 1 min and then washed with sterilized water. The pathogenic and endophytic strains of *B. sorokiniana* were cultured on PDA medium at 28 °C for seven days and adjusted to the same concentration of 10^5^ CFU/mL. Up to 200 μL of *B. sorokiniana* suspensions were inoculated onto wheat leaves, then incubated in an illumination incubator at 25 °C for seven days. Subsequently, the disease symptoms of wheat leaves were observed and recorded with a digital camera and optical microscope. The W-*B. sorokiniana*-infected wheat leaves (WB-1, WB-2, and WB-3), P-*B. sorokiniana*-infected wheat leaves (PB-1, PB-2, and PB-3), and sterilized water-treated wheat leaves (CK-1, CK-2, and CK-3) were collected and transcriptome-sequenced. A part of the infected tissues were cultured on PDA medium; the growing fungus was isolated and identified based on the morphological and biological characteristics. The cultivated P-*B. sorokiniana* at 28 °C for three days (samples A3-1 and A3-2) and seven days (A7-1 and A7-2), as well as cultivated pathogenic W-*B. sorokiniana* isolated from infected tissues on PDA medium for seven days (samples B7-1 and B7-2), was collected and transcriptome-sequenced [36]. The two *B. sorokiniana* strains were observed by scanning electron microscope (Hitachi S-3000N).

### 4.3. Total RNA Extraction and Sample Preparation for RNA-Seq

The total RNAs of infected wheat tissues and pathogenic and endophytic *B. sorokiniana* strains were extracted using an RNA extraction kit (Umagen, Guangzhou, China). The quality of the extracted RNAs was checked with a Nanodrop 2000 spectrophotometer (Thermo Fisher Scientific, Waltham, MA, USA). HPLC Agilent 2100 was used to detect the integrity of the total RNAs. The samples with high quality (RNA integrity number ≥6.0) were selected for transcriptome sequencing. The extracted total RNAs with an amount of 5.0 μg were resuspended in RNase-free water and stored at −80 °C until use. The extracted RNA samples were used for cDNA synthesis. The poly(A) mRNA was isolated using oligo-dT beads (Qiagen, Valencia, CA, USA). All mRNA was broken into short fragments (200 nt) using fragmentation buffer. First-strand cDNA was generated using random hexamer-primed reverse transcription, followed by the synthesis of the second-strand cDNA using RNase H and DNA polymerase I. The cDNA fragments were purified using a polymerase chain reaction (PCR) extraction kit. The purified fragments were ligated to sequencing adapters after poly(A) addition. The cDNA fragments (200 ± 25 bp) were purified and enriched via PCR to construct the final cDNA library by cDNA extraction from gels. The cDNA library was sequenced on the Illumina sequencing platform (Illumina HiSeq™ 2500, San Diego, CA, USA) using the paired-end (PE) technology within a single run, in which 100 bp PE reads were obtained. The original image process to sequences, base calling, and quality value calculation were performed using the Illumina GA Pipeline (version 1.6) [42,43]. All the sequencing processes of different *B. sorokiniana* samples were conducted in duplicates.

### 4.4. Assembly, Comparative Analysis, and Functional Annotation of the Transcriptome

Transcriptome sequencing was conducted using the Illumina Hi Seq™ 2500 platform. A PE 100 sequencing strategy was used to assemble all the transcriptomes of different samples. All the sequences were examined to ensure their accuracy. A Perl program was written to select clean reads by removing low-quality sequences (over 50% of the bases had qualities lower than 20 in one sequence), reads with over 5% N bases (bases unknown), and reads with adaptor sequences. Prior to assembly, adapters were clipped and low-quality bases were trimmed. Short sequences (<50 bp) were removed using a customized Perl program. The obtained high-quality sequences were deposited in the NCBI database and de novo assembled into contigs and transcripts. Transcripts with a minimum length of 200 bp were assembled and clustered using the software CLC NGS Cell under default parameters to reduce data redundancy. Searches were performed using local BLASTX programs against sequences in the NCBI nonredundant (Nr) protein and SwissProt databases. The e-value cutoff was 1 E−5 [44]. Unigenes were tentatively identified according to the top hits against known sequences. The resulting unigenes were used as references to determine the Gene Ontology (GO) and Clusters of Orthologous Group terms and were further analyzed by KEGG.

### 4.5. GO Classification and Pathway Enrichment Analyses

Differentially expressed genes (DEGs) were annotated based on the GO database using Blast2GO software 5.2.5 [45] according to their numerical orders in the Nr database to determine the main biological functions. Blast2GO is an all-in-one tool used for the functional annotation of (novel) sequences and the analysis of annotation data, which have been cited by other articles over 150 times. This tool is also a widely recognized GO annotation software. The GO annotations of each DEG were acquired. GO analysis was performed using the OmicShare tools, a free online platform for data analysis (http://www.omicshare.com/tools/Home/Soft/osgo). The GO enrichment analysis of functional significance terms in the GO database was conducted using a hypergeometric test to find significantly enriched GO terms in DEGs for comparison with the genome background. The Bowtie2 was used for the analysis of DEGs. edgeR 3.28.0 was used to perform the differential expression analysis. 

### 4.6. Comparative Expression Analysis

Reads that can be uniquely mapped into a gene were used to calculate the expression level. The gene expression level was measured by the number of uniquely mapped reads per kilobase of exon (RPKM). RPKM method eliminated the influences of different gene lengths and sequencing discrepancies on calculating gene expression. Therefore, the RPKM value can be directly used to compare the differences in gene expression among the samples. The fold changes of the unigene expression values, with *p*-values compared with each of the different samples, were used to report differential expression. Those with a *p*-value of <0.05 were considered significant differential expressions.

The expression patterns of all the obtained differential expression genes were discovered using the Short Time-series Expression Miner (v1.3.8) [46]. Genes were clustered according to their different expressions in samples. The DEGs that belonged to the same cluster exhibited similar expression patterns with one another. Therefore, clusters with specific expression patterns were selected and verified.

Genes with similar expression patterns typically indicate functional correlation. We performed cluster analysis of gene expression patterns using a clustering software cluster 3.0 and Java treeview software 1.1.6 [47,48,49].

### 4.7. Validation of the Gene Expression

The unigenes related to pathogenicity and defensive responses in different samples were validated via qRT-PCR to verify the quality of sequences assembled in this study. qRT-PCR was performed using a Mastercycler^®^ ep realplex system (Eppendorf, Hamburg, Germany) with SYBR Green (Invitrogen, Carlsbad, CA, USA) as the fluorescent dye, prepared according to the manufacturer’s instructions. Three independent biological repetitions were performed. First-strand cDNA was synthesized from 1 μg of total RNA with reverse transcriptase (Takara, Kyoto, Japan) and oligo(dT)15 primer. The resulting products were used as templates for qRT–PCR. The primers were designed with Primer Premier 5 according to the unigenes. Table S6 presents a list of the specific primers used for qRT–PCR. The qRT–PCR thermal cycling condition for all reactions was 95 °C for 1 min and 50 s, followed by 40 cycles of 95 °C for 10 s, 55 °C for 33 s, and 68 °C for 30 s. All reactions were conducted in biological triplicates. The glyceraldehyde 3-phosphate dehydrogenase gene was used as the reference gene. The obtained *C*_T_ values were used as the original data to calculate the relative expression levels of different genes to the histone gene via the 2^−^^Δ∆*C*T^ method [50,51].

### 4.8. The Metabolomics Analysis of the Two B. sorokiniana Strains

The two *B. sorokiniana* strains isolated from infected wheat tissues were cultivated for 7 days in 200 mL potato dextrose broth (PDB) medium and then collected, the fermentation liquids were extracted with methanol and concentrated by rotary evaporation. Each strain was cultivated in three 500-mL flasks, and the three concentrated fermentation liquids were loaded onto HPLC (Nexera X2 system, Shimazdu, Kyoto, Japan) with a C_18_ column (ZORBAX Eclipse Plus, Agilent, Santa Clara, CA, USA) and TOF-MS (Triple TOF 5600+, SCIEX, Foster, CA, USA). Different metabolites were eluted using a gradient with different concentrations of acetonitrile. The abundances of different compounds were calculated according to the peak areas. After the raw data were processed and rectified, the differential expression metabolites were identified through a series of statistical analyses.

## 5. Conclusions

In conclusion, this study is the first report on a comparative transcriptome combined with metabolome analysis between the two *B. sorokiniana* strains derived from *P. cablin* and wheat leaves, respectively, and the comparative transcriptomes of the two *B. sorokiniana* strain-infected wheat leaves were also firstly investigated. The *B. sorokiniana* isolated from wheat showed significantly stronger pathogenicity toward wheat than endophytic *B. sorokiniana* from *P. cablin.* The prediction of the significant differential unigenes and the qRT-PCR results confirmed that unigenes encoding pathogenicity protein LopB, cell wall-degrading enzymes (especially glycosyl hydrolase), and killer and Ptr necrosis toxin-producing related unigenes in W-*B. sorokiniana* played significant roles in its pathogenicity toward wheat. The comparative transcriptome analysis of the wheat infected with two *B. sorokiniana* strains indicated that the photosynthetic system and the cell wall of wheat leaves were impaired by W-*B. sorokiniana*, and the produced ROS caused damage to wheat cells. NAC transcription factors, hydrolyze enzyme inhibitors (including subtilisin-chymotrypsin inhibitor, serine protease inhibitor, and xylnase inhibitor), and peroxidases in wheat exerted enormous functions on the defensive responses of wheat to *B. sorokinina* infection. This research could lay a molecular foundation for the elucidation of the pathogenic mechanism of W-*B. sorokiniana* toward wheat, thus providing molecular clues for controlling wheat disease caused by W-*B. sorokiniana.*.

## Supplementary Materials

The following are available online at https://www.mdpi.com/1422-0067/20/23/6090/s1.
